# Plasma Level of ADAMTS13 or IL-12 as an Indicator of HBeAg Seroconversion in Chronic Hepatitis B Patients Undergoing m-ETV Treatment

**DOI:** 10.3389/fcimb.2020.00335

**Published:** 2020-07-24

**Authors:** Jiezuan Yang, Renyong Guo, Dong Yan, Haifeng Lu, Hua Zhang, Ping Ye, Linfeng Jin, Hongyan Diao, Lanjuan Li

**Affiliations:** ^1^State Key Laboratory for Diagnosis and Treatment of Infectious Diseases, National Clinical Research Center for Infectious Diseases, Collaborative Innovation Center for Diagnosis and Treatment of Infectious Diseases, The First Affiliated Hospital, College of Medicine, Zhejiang University, Hangzhou, China; ^2^Key Laboratory of Clinical in vitro Diagnostic Techniques of Zhejiang Province, Department of Laboratory Medicine, First Affiliated Hospital, College of Medicine, Zhejiang University, Hangzhou, China

**Keywords:** ADAMTS13, chronic hepatitis B, HBeAg seroconversion, IL-12, m-ETV

## Abstract

The ADAMTS13 (a disintegrin and metalloproteinase with a thrombospondin motif repeats 13) is a key factor involved in coagulation process and plays a vital role in the progression and prognosis of chronic hepatitis B (CHB) patients with antiviral treatment. However, there are few reports about the profile of plasma ADAMTS13 in CHB patients during entecavir maleate (m-ETV) treatment. One hundred two HBV e antigen (HBeAg)–positive CHB patients on continuous m-ETV naive for at least 96 weeks were recruited. Patients with liver cirrhosis were excluded using liver biopsies and real-time elastography. Plasma ADAMTS13 and interleukin 12 (IL-12) levels were evaluated at baseline and12, 24, 48, 72, and 96 weeks, respectively. The change of ADAMTS13 (ΔADAMTS13) and IL-12 (ΔIL-12) possesses a significant relationship in CHB patients with HBeAg seroconversion (SC) at 48-week m-ETV treatment (*p* < 0.001), but no significance in patients without SC. Furthermore, Cox multivariate analysis demonstrated that the change of ADAMTS13 (IL-12) is an independent predictor for HBeAg SC at week 96, and the area under the receiver operating characteristic curve for the ΔADAMTS13 (ΔIL-12) in CHB patients with 48-week m- ETV treatment is 0.8204 (0.8354) (*p* < 0.001, both) to predict HBeAg SC at week 96. The results suggested that higher increased ADAMTS13 and IL-12 after 48-week m-ETV treatment contributed to an enhanced probability of HBeAg SC, although the mechanism is undetermined. Quantification of ADAMTS13 (IL-12) during m-ETV treatment may help to predict long-term HBeAg SC in CHB patients.

## Introduction

Hepatitis B is a worldwide disease caused by hepatitis B virus (HBV) infection. There are approximately 290 million HBV carriers worldwide. More than 700,000 people die annually due to chronic hepatitis B (CHB) complications, including cirrhosis and liver cancer (Trepo et al., 2014; Lumley et al., 2018). The extensive application of hepatitis B vaccine significantly decreases the incidence rate of subjects newly infected with HBV. Nucleotide analogs (NUCs) can effectively postpone the disease progression of subjects with chronic HBV infection. However, some CHB patients need long-term NUC treatment (Wei and Kao, 2017), and antiviral resistance remains an important challenge for long-term CHB therapy (Kim et al., 2014). Suppression of HBV could delay the progression of CHB patients and restore the immune control of HBV DNA replication (European Association for the Study of the Liver, 2009).

The entecavir (ETV) is one of the most potent nucleoside analogs with a high genetic barrier to resistance and a favorable safety profile during long-term treatment of patients with CHB (Jia et al., 2017; Suzuki et al., 2019). Entecavir maleate (m-ETV) tablet is a maleate compound of ETV; with oral administration, m-ETV is transformed into ETV in human body, giving full play to its strong resistance. Moreover, the ingredient, animal toxicity, pharmacodynamics, and pharmacokinetics of m-ETV are similar to those of ETV (Xu et al., 2017b). In the anti-HBV treatment, HBV e antigen (HBeAg) seroconversion (SC) is a critical biomarker for sustaining HBV suppression and postponing disease progression (Xing et al., 2017; Wang et al., 2018) and means a favorable outcome of CHB patients with antiviral treatment. There are many reports involved in the antiviral properties and curative effect of ETV (Lam et al., 2017; Suzuki et al., 2019; van Campenhout et al., 2019), including our report (Guo et al., 2016). However, there are only seldom studies on the surrogate (biomarker) that could be used to predict the HBeAg SC of CHB patients during m-ETV treatment (van Campenhout et al., 2019).

In our previous report, we found that an increasing ADAMTS13: AC after 1 year of ETV treatment was associated with higher SC of CHB patients, which could predict SC of CHB with 5-year ETV treatment (Guo et al., 2019). Interleukin 12 (IL-12) is a heterodimeric cytokine produced by activated antigen-presenting cells, such as dendritic cells, macrophages, and natural killer (NK) cells. Elevation in IL-12 levels may be a factor to promote HBeAg SC of CHB patients (He et al., 2012). However, there are few reports about the IL-12 as diagnostic marker to predict HBeAg SC of CHB patients during antiviral treatment.

In this study, we measured the plasma dynamic levels of ADAMTS13 protein and IL-12 in serial samples of CHB patients who are HBeAg-positive (HBeAg^+^) and underwent up to 2 years of m-ETV treatment. The aim of the study was to determine the kinetic profile of plasma ADAMTS13 level and IL-12 of HBeAg^+^ CHB patients with m-ETV treatment and to identify that the factors could be used to predict HBeAg SC for CHB patients undergoing long-term antiviral treatment. Furthermore, this result would contribute to determine that ADAMTS13 and IL-12 are targets for treating patients with chronic HBV infection (Uemura et al., 2010; Schurich et al., 2013).

## Materials and Methods

### Subjects

The subjects of the study included 121 consecutively m-ETV treatment–naive CHB patients from the Department of Infectious Diseases of the First Affiliated Hospital of Zhejiang University from March 2010 to October 2015. The diagnostic standard of enrolled CHB subjects was according to the Asian-Pacific clinical practice guidelines on the management of hepatitis B (Sarin et al., 2016). Patients were treated with oral 0.5 mg/d m-ETV (Chia Tai Tianqing Pharmaceutical Co., Ltd., Jiangsu, China) for more than week 96; patients were regularly followed up. In addition, all the patients enrolled had the same inclusion and exclusion criteria: 20–50 years of age, HBsAg-positive for at least 6 months, HBeAg^+^ and hepatitis B e antibody (HBeAb) negative (HBeAb^−^). Patients receiving any antiviral treatment in the previous 6 months before screening, with coinfection or superinfection of hepatitis A virus, hepatitis C virus, hepatitis D virus, hepatitis E virus, Epstein-Barr virus, or cytomegalovirus, were excluded. The enrolling and exclusion criteria for patients were also as those previously reported (Guo et al., 2016; Xu et al., 2017a).

Patient follow-up and venous blood drawn were performed at 12-week intervals during the first half year and 24-week intervals thereafter for clinical assessments including liver function biochemical tests, hematologic examinations, and measurements of serological hepatitis B markers and HBV DNA levels. The study was approved by the ethics committee of the First Affiliated Hospital of Zhejiang University. Informed consent was obtained from each patient included in the study, and the study protocol conforms to the ethical principles of the Declaration of Helsinki.

### Clinical and Laboratory Evaluation

Plasma albumin (ALB), alanine aminotransferase (ALT), aspartate transaminase, and other conventional biochemical indicators were determined using a Hitachi 7600 analyzer (Hitachi Ltd., Tokyo, Japan) at local laboratories. HBsAg, HBeAg, anti-HBs Ab, anti-HBe Ab, and anti-HBc Ab were measured by enzyme immunoassay (AxSYM; Abbott Laboratories, Abbott Park, IL, USA) or chemiluminescence immune assay. Plasma HBV DNA levels were assayed using the COBAS TaqMan (Roche Diagnostics, Indianapolis, IN, USA) as our previous report (Guo et al., 2016), with the lower limit of detection of 20 IU·mL^−1^.

Plasma von Willebrand factor (VWF) and ADAMTS13 protein level were determined using commercial enzyme-linked immunosorbent assay (ELISA) kits (Abcam, Cambridge, UK) according to the manufacturer's instructions. The ELISA kit (Abcam) was also used to detect the plasma level of IL-12. Before detection, the plasma was diluted to find the optimal concentration for determining the real levels of VWF, ADAMTS13, and IL-12, respectively.

In order to separate liver cirrhosis from patients with CHB infection, all the 121 CHB patients were subjected to liver stiffness assessed via real-time elastography (RTE) before m-ETV treatment, among whom 41 patients had a liver biopsy. Only non-cirrhotic patients with METAVIR score (F0~F3) or liver stiffness index (≤12.4 kPa, if the METAVIR score was unavailable) were enrolled in the study.

### Statistical Analysis

All statistical tests were performed using SPSS version 21.0 (SPSS Inc., Chicago, IL, USA). Continuous variables are expressed as mean ± standard deviation (SD), or median (25th−75th percentile). Plasma HBV DNA and HBsAg levels are expressed in logarithmic scale (log_10_, l g). Differences in mean and median values were assessed by using an unpaired *t*-test or Mann–Whitney *U*-test, respectively. Categorical variables were compared using Pearson χ^2^-test or Fisher exact test as appropriate. We used the Spearman coefficient to assess the correlation between ΔADAMTS13: Ag and ΔIL-12 level. Multivariate analysis logistic regression and Cox proportional hazards were performed to examine the influence of the variables (ΔADAMTS13: Ag, ΔIL-12) on HBeAg SC.

The cutoff points for continuous variables were determined by receiver operating characteristic (ROC) curve analysis. Time-to-event curves were plotted with the Kaplan–Meier method, and comparisons were made using a log-rank test. Overall survival was calculated based on the date of initiation of m-ETV treatment until the date of last follow-up or the date of the occurrence of HBeAg SC. A two-tailed *p* < 0.05 was considered statistically significant for all tests. All figures were produced with the software (GraphPad Prism 7.0, San Diego, CA, USA); moreover, the Sankey diagram (R program package) was used to show the contributing rate of several variables on the HBeAg SC of CHB patients during m-ETV treatment.

## Results

**Figure 1:**
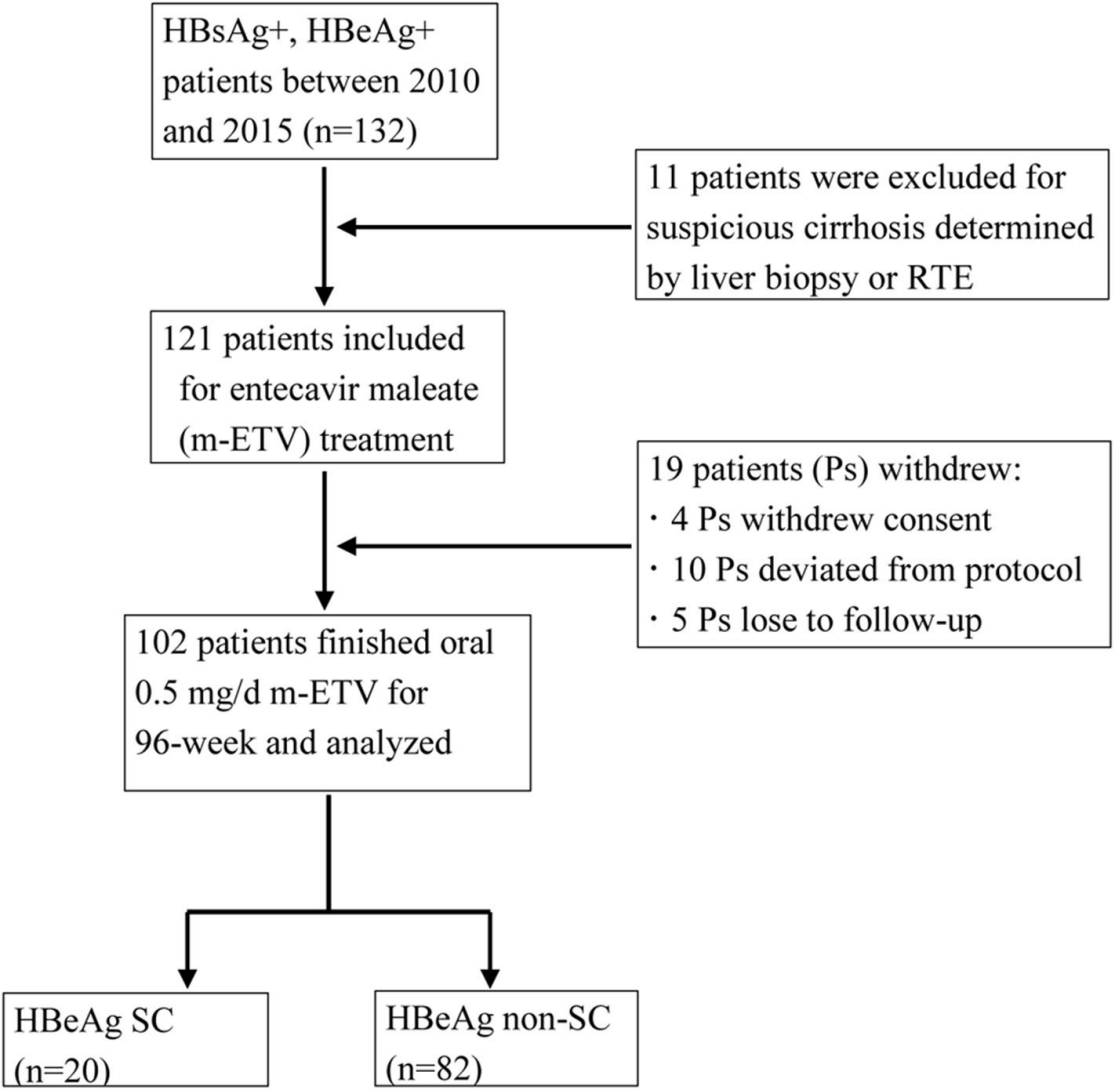
Flowchart of the CHB patients included in the study.

A total of 102 positive HBeAg CHB patients (102/121 [84.3%]) completed 2-year (96 weeks) m-ETV–naive treatment, and 19 patients (15.7%) withdrew prematurely, including five lost follow-up. More details are shown in Figure 1. Additionally, in terms of clinical manifestations, there are no other liver-related complications or hepatitis flares discovered throughout the entire 96-week m-ETV treatment. Minor complications included mild fever and rash, which could be quickly recovered after symptomatic treatment.

### Clinical Characteristics of Patients and Comparisons Between CHB Patients With and Without HBeAg SC

Finally, there are 102 HBeAg^+^ CHB patients who completed 96-week antiviral treatment, among whom 20 (19.6%) had undergone HBeAg SC, the rest (80.4%) without HBeAg SC at this time point. Moreover, all 102 subjects who continued were subjected to antiviral treatment after this time point. At baseline clinical characteristics, there is no significant difference between patients with HBeAg SC and without SC, including the levels of IL-12 and ADAMTS13; the latter was consistent with the significant correlations of our previous report (Guo et al., 2019). Furthermore, the detail clinic characteristics of 102 involved CHB patients are shown in Table 1, and the ADAMTS13 and IL-12 concentrations of each CHB patient at baseline (before m-ETV treatment) are also shown (Figure S1).

**Table 1:** Baseline characteristics of CHB patients with HBeAg seroconversion (SC) or without HBeAg SC undergoing 2 years of entecavir maleate treatment.

**Variables**	**All subjects**	**With HBeAg SC**	**Without SC**	***p*^a^**
Patients (*n*)	102	20	82	—
Age (years)	37.95 ± 8.54	39.50 ± 7.70	37.57 ± 8.73	0.413
Gender (M/F)	87/15	16/4	71/11	0.486^b^
Body weight (kg)	56.91 ± 8.06	56.90 ± 8.12	56.91 ± 8.09	0.794
ALT (U/L)	222.86 ± 76.79	232.60 ± 78.05	222.49 ± 76.77	0.552
TBi (μmol/L)	18.50 (17.00, 23.00)	18.00 (16.75, 23.25)	19.50 (17.00, 22.75)	0.867
ALB (g/L)	44.08 ± 7.05	44.54 ± 6.65	43.96 ± 7.18	0.787
WBC (×10^9^/L)	4.80 ± 1.30	4.89 ± 1.36	4.78 ± 1.30	0.670
HBV DNA (log10, copies/mL)	7.82 (6.47, 9.06)	8.15 (7.41, 9.06)	7.79 (6.36, 9.08)	0.383
HBsAg (log10 IU/mL)	4.24 (3.42, 4.95)	4.25 (3.56, 4.74)	4.24 (3.35, 5.16)	0.990
HBeAg (PEIU/mL)	2.79 (1.89, 4.28)	2.56 (2.16, 3.48)	2.81 (1.89, 4.45)	0.372
ADAMTS13: AC (U/mL, mean ± SD)	7.88 ± 1.27	7.82 ± 2.14	7.90 ± 0.97	0.882^c^
VWF: Ag (U/L)	15.75 (11.49, 17.88)	15.41 (12.71, 18.97)	15.98 (11.42, 17.83)	0.693
IL-12 (ng/mL)	4.50 (3.30, 6.43)	4.25 (3.08, 5.75)	4.80 (3.30, 6.48)	0.519

Clinical and biochemical characteristics of the study patients; values are expressed as median (Q1–Q3), number, or mean ± standard deviation. p-values for differences of baseline variables between with and without HBeAg seroconversion of CHB are from

a *Mann-Whitney U-test*,

b *χ^2^-test*,

c *two-tailed independent-samples t-test*.

*VWF Ag, VWF antigen; ADAMTS13: AC, ADAMTS13 activity; TBi, Total bilirubin*.

**Figure 2:**
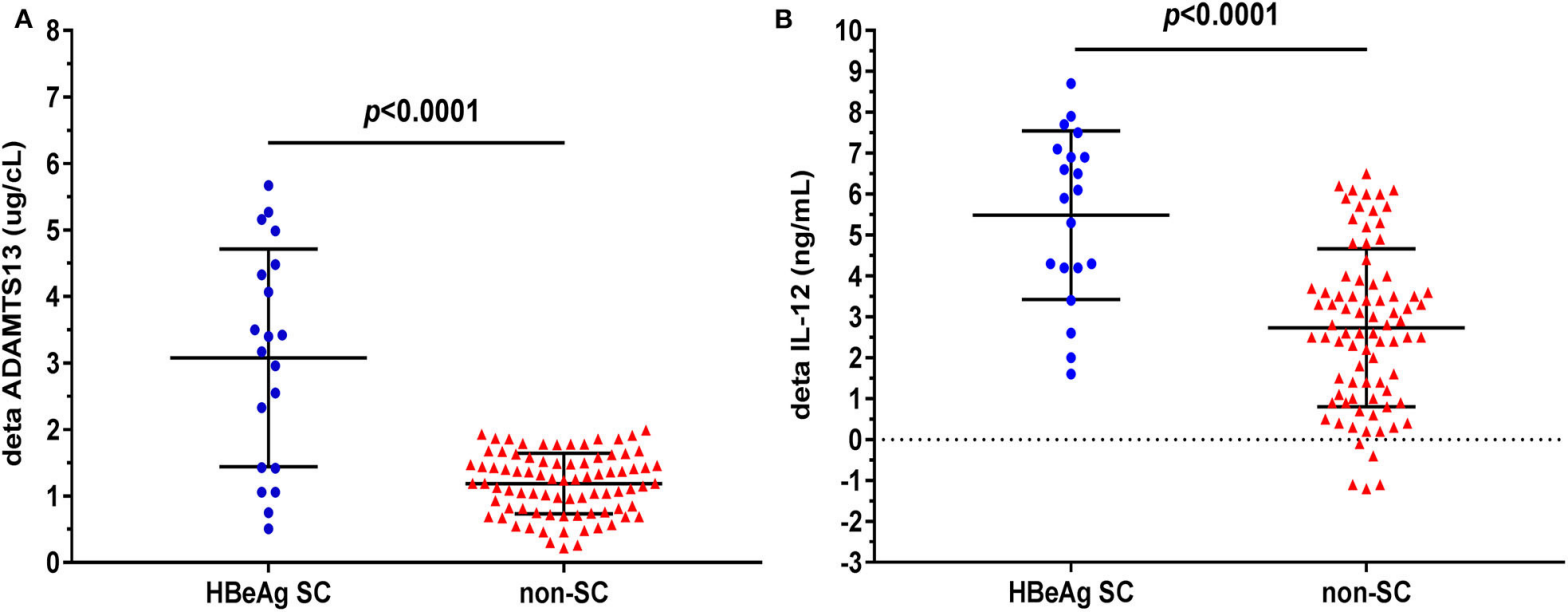
Difference of plasma delta (Δ) ADAMTS13 or delta (Δ) IL-12 levels between CHB patients with HBeAg seroconversion (SC) and without. ΔADAMTS13 (change after m-ETV treatment of 48 weeks) **(A)** levels are significantly higher in CHB patients with HBeAg SC than in those without SC; similarly, plasma ΔIL-12 **(B)** levels are significantly higher in patients with HBeAg SC. Data are expressed as scatter plots, in which the middle of the black solid line is the median, and the up and down horizontal lines represent the 25th and 75th percentiles, respectively.

As shown in Figure 2, however, patients undergoing HBeAg SC exhibited significantly elevated plasma ADAMTS13 than patients without HBeAg SC at 48 weeks of m-ETV treatment [3.08 ± 1.64, *n* = 20 vs. 1.19 ± 0.46 μg/cL, *n* = 82, *p* < 0.0001]; simultaneously, there is significantly increased IL-12 levels in patients with SC than that of patients without HBeAg SC [5.49 ± 2.06 vs. 2.73 ± 1.93 ng/mL, *p* < 0.0001].

### Relationship Between ADAMTS13 or IL-12 and Clinical Variables

Before treatment (at baseline), the ADAMTS13 level is positively or negatively correlated with clinical variables that were determined at the present study. However, there was no significance among them (Table 2). Simultaneously, we found there was no significant relationship between IL-12 and clinical variables at baseline, although IL-12 level is positively or negatively correlated with clinical variables determined (Table 3).

**Table 2:** Relation between the baseline levels of plasma ADAMTS13 activity and clinical variables in CHB patients.

**Variables**	**ADAMTS13: AC expression levels**	
	**Spearman correlation**	***p***
Age (years)	−0.040	0.692
Gender (M/F)	−0.104	0.297
Body weight (kg)	−0.031	0.760
ALT (U/L)	−0.101	0.314
TBi (μmol/L)	0.112	0.263
ALB (g/L)	−0.065	0.516
WBC (×10^9^/L)	0.055	0.583
HBV DNA (log_10_, copies/mL)	0.039	0.699
HBsAg (log10 IU/mL)	−0.085	0.394
HBeAg (PEIU/mL)	0.145	0.145
VWF: Ag (U/L)	0.036	0.717
IL-12 (mg/mL)	0.096	0.337

**Table 3:** Relation between the baseline levels of plasma IL-12 and clinical variables in CHB patients.

**Variables**	**IL-12 expression levels**	
	**Spearman correlation**	***p***
Age (years)	−0.150	0.133
Gender (M/F)	−0.109	0.275
Body weight (kg)	−0.119	0.232
ALT (U/L)	0.052	0.601
TBi (μmol/L)	−0.186	0.061
ALB (g/L)	0.170	0.087
WBC (×10^9^/L)	−0.153	0.126
HBV DNA (log_10_, copies/mL)	−0.147	0.139
HBsAg (log10 IU/mL)	0.054	0.591
HBeAg (PEIU/mL)	0.031	0.758
ADAMTS13: AC (U/L)	0.096	0.337
VWF: Ag (U/L)	−0.081	0.420

However, during the course of m-ETV treatment, there was a significant positive correlation between changes in plasma ADAMTS13 and the IL-12 levels in seroconverting patients (*r*^2^ = 0.9063, *p* = 0.0034; Figure S2A), and a similar relationship was found in patients without HBeAg SC (*r*^2^ = 0.891, *p* = 0.0029; Figure S2B).

### Increasing ADAMTS Is Positive Relative to Increasing IL-12 in the Group With SC

**Figure 3:**
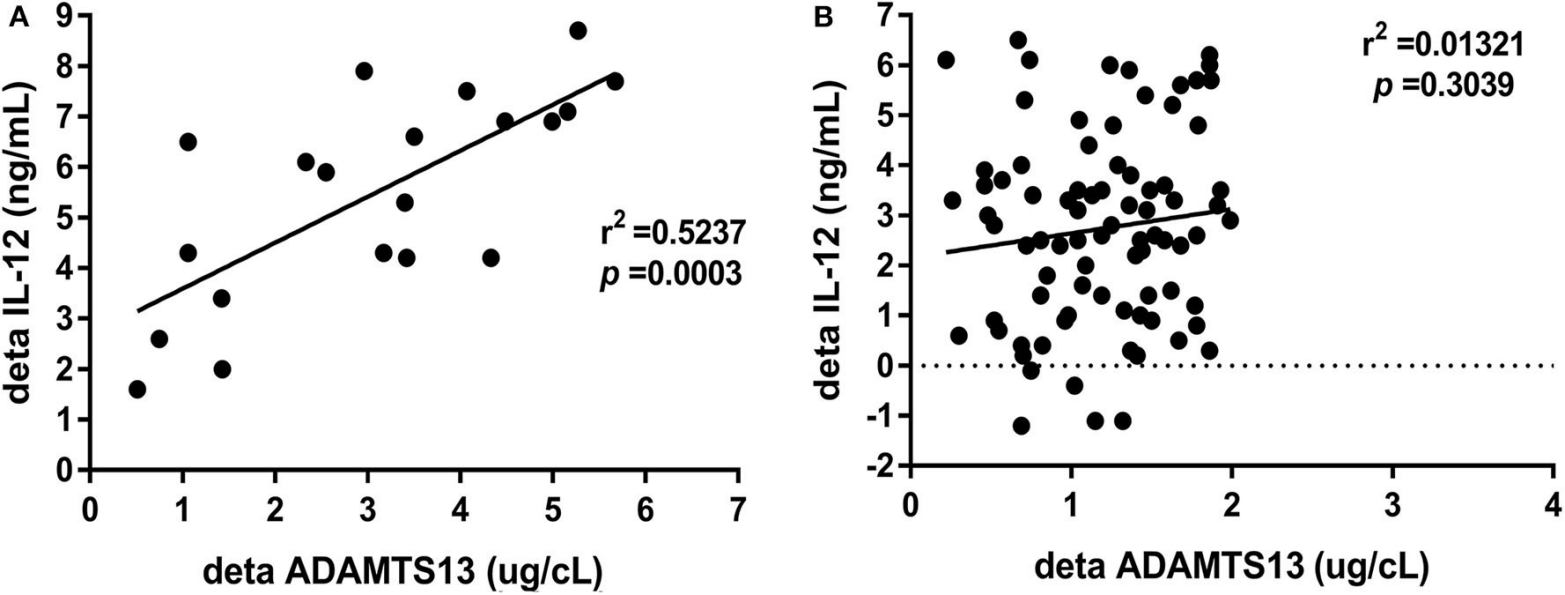
Relationship between the ΔADAMTS13 and ΔIL-12 levels of CHB patients with HBeAg SC **(A)** or without SC **(B)** at m-ETV treatment of 96 weeks. Delta (Δ) expressed as the change after m-ETV of 48 weeks.

The relationship between plasma ΔADAMTS (change after m-ETV treatment of 48 weeks) and ΔIL-12 levels was analyzed in 102 CHB patients using Spearman correlation analysis. There was a statistically positive correlation in SC group (*r*^2^ = 0.5237, *p* = 0.0003; Figure 3A), but there was no significant correlation in CHB patients without HBeAg SC during the 48-week m-ETV treatment (*r*^2^ = 0.0132, *p* = 0.3039; Figure 3B).

### Predictive Values of ΔADAMTS13 and ΔIL-12 in HBeAg SC of Patients During m-ETV Treatment

Cox univariate regression analysis demonstrated that those patients with higher ΔADAMTS13 and ΔIL-12 after 1 year (48 weeks) of m-ETV treatment had a significantly greater probability of HBeAg SC (*p* = 0.000, both). Based on multivariate analysis, the ΔADAMTS13 levels after 1 year of m-ETV treatment [hazard ratio (HR) = 2.614, 95% confidence interval (CI) = 1.640–4.166, *p* = 0.000] and the ΔIL-12 after 1 year of m-ETV treatment (HR = 1.716, 95% CI = 1.274–2.312, *p* = 0.000) were independent predictors of HBeAg SC at year 2 (Table 4).

**Table 4:** Univariate and multivariate analyses for HBeAg seroconversion in CHB patients with 2 years of entecavir maleate treatment.

**Variables^a^**	**HR (95% CI)**	***p***
**UNIVARIATE ANALYSIS**		
Age (years)	1.024 (0.971–1.081)	0.379
Gender (M/F)	0.658 (0.220–1.967)	0.453
Body weight (kg)	0.990 (0.938–1.046)	0.726
ALT (U/L)	1.001 (0.996–1.007)	0.628
TBi (μmol/L)	1.005 (0.903–1.119)	0.929
ALB (g/L)	1.009 (0.947–1.074)	0.786
WBC (×10^9^/L)	1.078 (0.764–1.522)	0.669
HBV DNA (log_10_, copies/mL)	1.166 (0.862–1.578)	0.319
HBsAg (log_10_ IU/mL)	1.005 (0.659–1.533)	0.981
HBeAg (PEIU/mL)	0.830 (0.577–1.195)	0.317
ΔADAMTS13: AC (U/mL)	2.168 (1.694–2.776)	0.000
VWF: Ag (U/L)	1.026 (0.911–1.156)	0.672
ΔIL-12 (ng/mL)	1.768 (1.397–2.225)	0.000
**MULTIVARIATE ANALYSIS**		
ΔADAMTS13: AC (U/mL)	1.816 (1.409–2.341)	0.000
ΔIL-12 (ng/mL)	1.578 (1.241–2.007)	0.000

a *Clinical characteristic at baseline, delta (Δ) expressed as the change after ETV maleate of 1 year. HR, hazard ratio; CI, confidence interval*.

**Figure 4:**
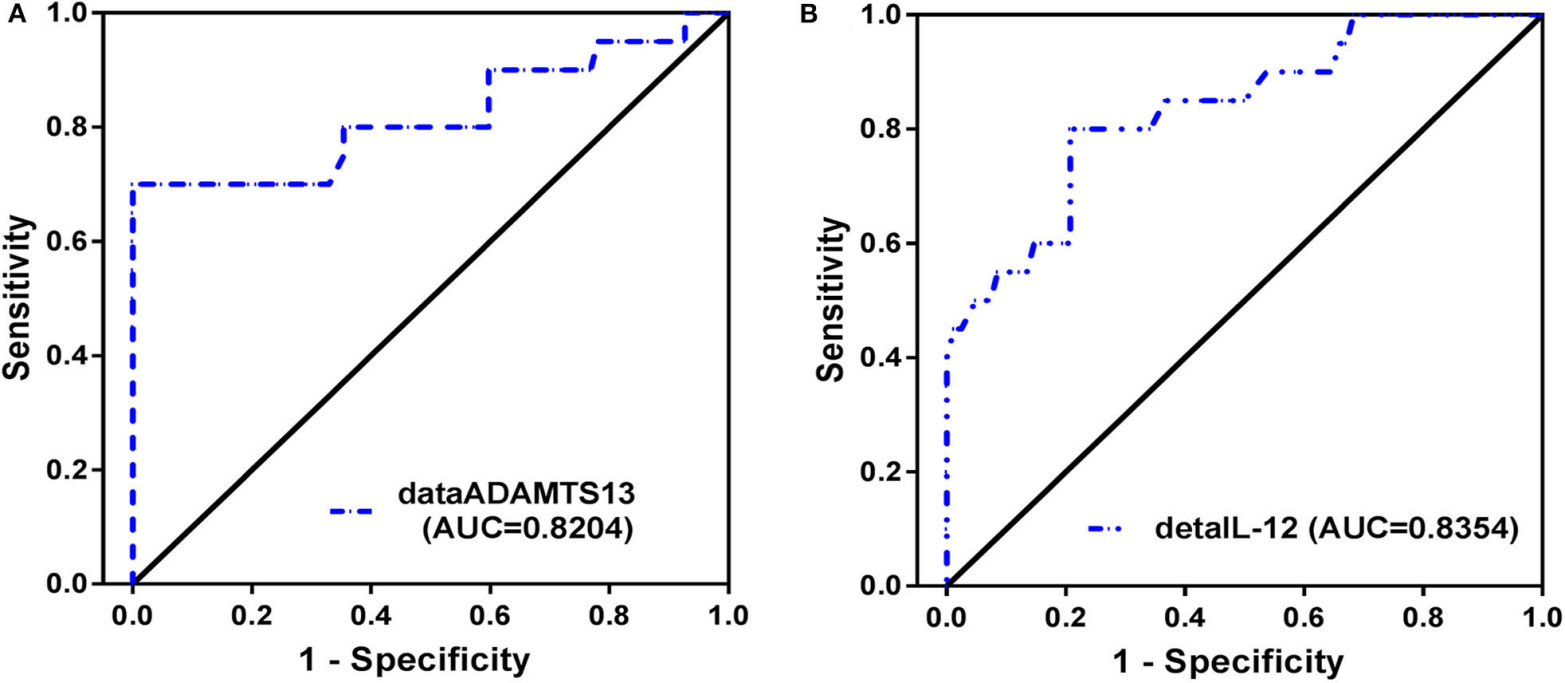
Receiver operating characteristic (ROC) curves. The ROC curves of the Δ (delta) ADAMTS13 **(A)**, ΔIL-12 **(B)** after 1 year of m-ETV treatment for separating HBeAg SC from non-SC in HBeAg (+) CHB patients with 96 weeks of continuous m-ETV treatment.

We further performed ROC analysis to assess the capacity of levels of ΔADAMTS13 and ΔIL-12 after 1 year of m-ETV treatment for predicting a year 2 HBeAg SC. The AUC values were consistently high for ΔADAMTS13 (AUC = 0.820, 95% CI = 0.688–0.953) and ΔIL-12 (AUC = 0.835, 95% CI = 0.733–0.938; Figures 4A,B), and the differences observed between them were significant (*p* < 0.001, both). Moreover, with respect to the ΔADAMTS13, the optimal cutoff value for the prediction of the patient undergoing HBeAg SC was 2.16 μg/cL (sensitivity = 70.0% and specificity = 100.0%), and the positive and negative predictive values are 100.0 and 93.2%, respectively. Simultaneously, to the ΔIL-12, the cutoff value was 4.10 ng/mL (sensitivity = 80.0% and specificity = 79.3%), with positive and negative predictive values 48.5 and 94.2%, respectively. Additionally, the sensitivity and specificity for predicting HBeAg SC can be vividly found at Sankey diagram (Figure S3).

**Figure 5:**
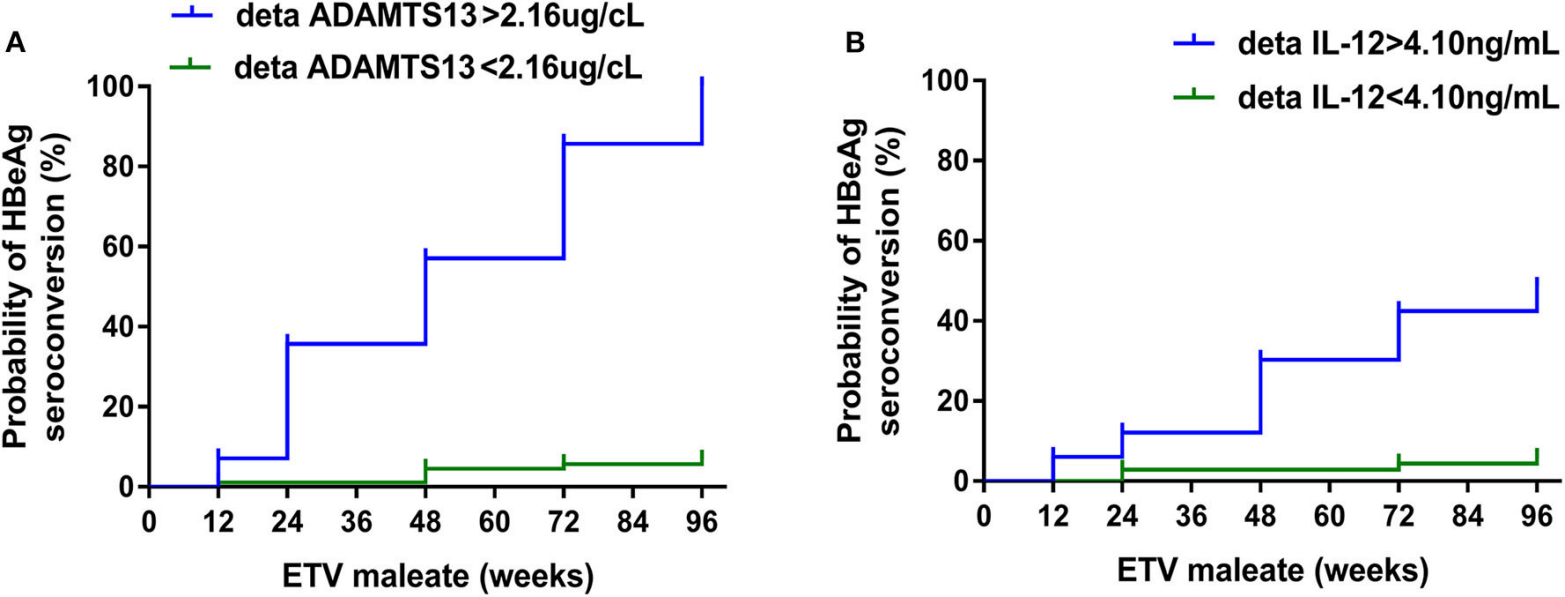
Probability of HBeAg SC for all 102 CHB patients (96 weeks) stratified by cutoff value of ΔADAMTS13 **(A)** or **(B)** ΔIL-12 levels after a 48-week m-ETV treatment.

Furthermore, when the cutoff value of 2.16 μg/cL for ΔADAMTS13 after 1 year of m-ETV treatment was applied to assess the cumulative rates of HBeAg SC, patients with ΔADAMTS of >2.16 μg/cL were found to achieve significantly higher probability of HBeAg SC than patients with ΔADAMTS of <2.16 U/mL at 48-week m-ETV treatment (HR = 24.17, 95% CI = 5.147–113.5, *p* < 0.001). Additionally, for ΔIL-12 (cutoff value, 4.10 ng/mL), the similar result was obtained (HR = 10.16, 95% CI = 3.839–26.91, *p* < 0.001; Figures 5A,B).

## Discussion

The present cross-sectional observational study expressed that higher increasing ADAMTS13 and IL-12 levels, but not the baseline levels of ADAMTS13 and IL-12, were significantly and positively associated with the prevalence of HBeAg SC in CHB patients during 96-week m-ETV treatment, suggesting higher increasing plasma ADAMTS13 (IL-12) level as a definite probability for HBeAg SC, independent of other factors, such as HBV DNA, HBsAg, HBeAg, and VWF, although the VWF level is closely related to ADAMTS13 concentration in the peripheral blood of CHB patients.

There are many literatures about the pathogenesis of patients with chronic HBV infection, and the imbalance of a coagulation system also plays an important role in CHB pathogenesis (Saray et al., 2012; Chang and Liu, 2016; Fu et al., 2019). Von Willebrand factor is a member of the main proteins involved in hemostasis and thrombosis in the body. The size of VWF polymer is closely linked to its function; the larger molecular weight is, the stronger its binding ability to collagen and platelets will be. ADAMTS13 plays an antithrombotic role by lysing VWF polymers. ADAMTS13 is mainly synthesized and secreted by hepatic stellate cells and vascular endothelial cells and is slightly expressed in all tissues and organs (Asada et al., 2018). Ley et al. found that ADAMTS13 can inhibit inflammatory by cleaving the VWF with very large molecular weight (UL-VWF) and by preventing excessive white blood cell (WBC) chemotaxis to veins and WBC adhesion and extravasation (Ley et al., 2007). Therefore, the ADAMTS13 is involved in the process of inflammation in patients with CHB provoked by HBV (Manea et al., 2010). However, clinical implications and possible treatment perspectives for the ADAMTS13 in the liver remain not fully understood.

At the present study, there is no difference between the CHB patients with HBeAg SC and without in the baseline levels of ADAMTS13, IL-12, which is consistent with the GEO database (Figure S4). However, the plasma ΔADAMTS (change after m-ETV treatment of 48 weeks) and ΔIL-12 levels could predict the HBeAg SC of patients at the 96-week m-ETV treatment. Furthermore, the ΔADAMTS13 level is an independent factor determining probability of HBeAg SC, excluding other factors, such as HBV DNA, HBsAg, and HBeAg, which was confirmed by Cox univariate (multivariate) regression analysis, and ΔIL-12 has similar predictive ability for HBeAg SC.

Interleukin 12 is a cytokine that is naturally produced by dendritic cells, macrophages, neutrophils, and human B-lymphoblastoid cells (NC-37) in response to antigenic stimulation and is still a promising candidate for tumor immunotherapy (Lasek et al., 2014). It stimulates the production of interferon γ (IFN-γ) and tumor necrosis factor α from T cells and NK cells and reduces IL-4–mediated suppression of IFN-γ (Orange, 2008). The favorable outcome of CHB patients (with or without antiviral treatment) is closely related to restore the immunity and control of HBV DNA replication (Wu et al., 2015). Additionally, the IL-12 level is highest in acute hepatitis B accompanied by HBeAg SC, and the IL-12 levels may be an opportunity to be given antiviral treatment for immune-tolerance carriers (He et al., 2012; Tavakolpour et al., 2017); however, in woodchucks model, the IL-12 induces a strong immunosuppressive reaction in the liver of chronic WHV carriers that counteracts the antiviral effect of the treatment (Otano et al., 2012). Therefore, the application of IL-12 for treating chronic HBV infection should be confirmed in future study.

Interleukin 12 comprised a bundle of four α helices. It is a heterodimeric cytokine encoded by two separate genes, IL-12A (p35) and IL-12B (p40) (Venetz et al., 2016). The IL-12 p70 is a combination of IL-12A (p35) and IL-12B (p40) and is an activity unit of IL-12. Here, the relationship between the change levels of ADAMTS13 and IL-12 is a significantly positive correlation in CHB patients with HBeAg SC, but not in patients without HBeAg SC. This finding will contribute to predicting the HBeAg SC or not in CHB patients in the following m-ETV treatment.

During anti-HBV treatment, HBeAg SC usually is surrogate of favorable prognosis for CHB patients with positive HBeAg. In the past, we reported that plasma ADAMTS13 activity was associated with HBeAg SC in CHB patients during 5 years of ETV treatment (Guo et al., 2019). In the present study, we find the change of ADAMTS13 or IL-12 is closely associated with HBeAg SC for CHB patients during m-ETV treatment. To our best knowledge, it is primarily time to find a biomarker being relative to HBeAg SC of CHB patients with m-ETV treatment. Additionally, ROC curves were plotted to define the optimal cutoff values of the ΔADMATS13 (change after m-ETV treatment of 48 weeks) and ΔIL-12 for discriminating a week 96 HBeAg SC in HBeAg^+^ patients. They all achieve a significantly higher probability of HBeAg SC. Thus, we found an optional biomarker for discrimination CHB patients with or without undergoing HBeA SC, but should be further confirmed with more cases in future research. Moreover, increasing size of enrolled cases may increase the number of SC patients, which could provide more clear-cut tendency for the ADAMTS13 or IL-12 discrimination of SC from non-SC populations.

There are some limitations to our study; first, the reduction of ADAMTS13 level is not only a biomarker of disease severity, but also an independent indicator of organ dysfunction and poor prognosis (Gando, 2010), which implies the key role of ADAMTS13 involved in the pathogenic mechanisms. However, we have not investigated which signal pathway ADAMTS13 is involved in and plays the process of HBeAg SC. Second, the plasma ADAMTS13 level is related to liver cirrhosis (Uemura et al., 2008); the METAVIR scoring system (liver biopsy, F0~F3) is the gold standard; only some patients subjected to liver biopsy, although all patients underwent liver stiffness assessed via RTE, and RTE is non-invasive and accurately stage hepatic fibrosis (Chen et al., 2017). Third, the plasma ADAMTS13: activity (AC) and antigen should be separately measured for getting precise results. In the study, we detected only the ADAMTS13 protein level according to the operational direction, although Uemura et al. (2008) reported that both plasma ADAMTS13: AC and antigen levels decreased with increasing severity of cirrhosis. Finally, it still requires further investigation whether the conclusions can be generalized to other cohorts in different countries with different HBV genotype infections, because only the Chinese patient populations were enrolled in our study.

## Conclusion

In summary, the higher increase of ADAMTS13 (IL-12) in CHB patients at 48 weeks of m-ETV treatment could predict HBeAg SC at a 96-week m-ETV treatment, although future studies should be made to confirm the role of these cytokines in the pathogenesis of HBeAg SC. Moreover, supportive therapies of ADAMTS13 (IL-12) supplementation may be contributed to favorable outcomes of CHB patients by improving intrahepatic microcirculatory disturbance, which would be a promising study.

## Data Availability Statement

The datasets analyzed in this article are not publicly available. Requests to access the datasets should be directed to yangyan@zju.edu.cn.

## Ethics Statement

The studies involving human participants were reviewed and approved by The Ethics Committee of the First Affiliated Hospital of Zhejiang University. The patients/participants provided their written informed consent to participate in this study.

## Author Contributions

JY contributed to the study design, experiments, writing the initial draft, and revising the manuscripts. RG and DY collected the clinical data and helped to perform some experiments. HL and HZ assisted in experimental design and help to data collection. PY and LJ participated in the study design and liver cirrhosis aided diagnosis. HD and LL contributed to the study coordination, technical issues, and revision of the manuscript. All authors read and approved the final manuscript.

## Conflict of Interest

The authors declare that the research was conducted in the absence of any commercial or financial relationships that could be construed as a potential conflict of interest.

## Supplementary Material

Figure S1 Comparison between CHB patients with and without HBeAg seroconversion in plasma ADAMTS13 and IL-12 levels during m-ETV treatment. Plasma ADAMTS13 **(A)** baseline levels are no significantly difference in those CHB patients with or without HBeAg SC, similarly, the IL-12 **(B)** baseline levels are no significantly difference either.

Figure S2 Association between plasma ADAMTS13 and IL-12 levels in HBeAg seroconverting **(A)** and non-seroconverting **(B)** patients during m-ETV treatment.

Figure S3 Sankey diagram of predicting HBeAg seroconversion. The variables of ΔADAMTS13, ΔIL-12, DNA (lg), HBsAg (lg), and HBeAg seroconversion (Yes, No) were put together to form Sankey diagram, in which variables could vividly present their contributing rate for SC in CHB patients during m-ETV treatment. The X-axis (left vertical numbers) shows the approximate number of CHB patients.

Figure S4 Expression of ADAMTS13 **(A)** or IL-12 **(B)** levels between patients with and without HBeAg seroconversion (SC). The data is from the GEO dataset (GSE54747), and was grouped into two groups (HBeAg SC and non-SC HBeAg) according to the message provided. Dataset (https://www.ncbi.nlm.nih.gov/geo/query/acc.cgi?acc=GSE54747).
