# Supplementary figures and images for: Plasma Level of ADAMTS13 or IL-12 as an Indicator of HBeAg Seroconversion in Chronic Hepatitis B Patients Undergoing m-ETV Treatment
Source: Front Cell Infect Microbiol. 2020 Jul 24;10:335. doi: 10.3389/fcimb.2020.00335 (PMC7393286; doi:10.3389/fcimb.2020.00335)

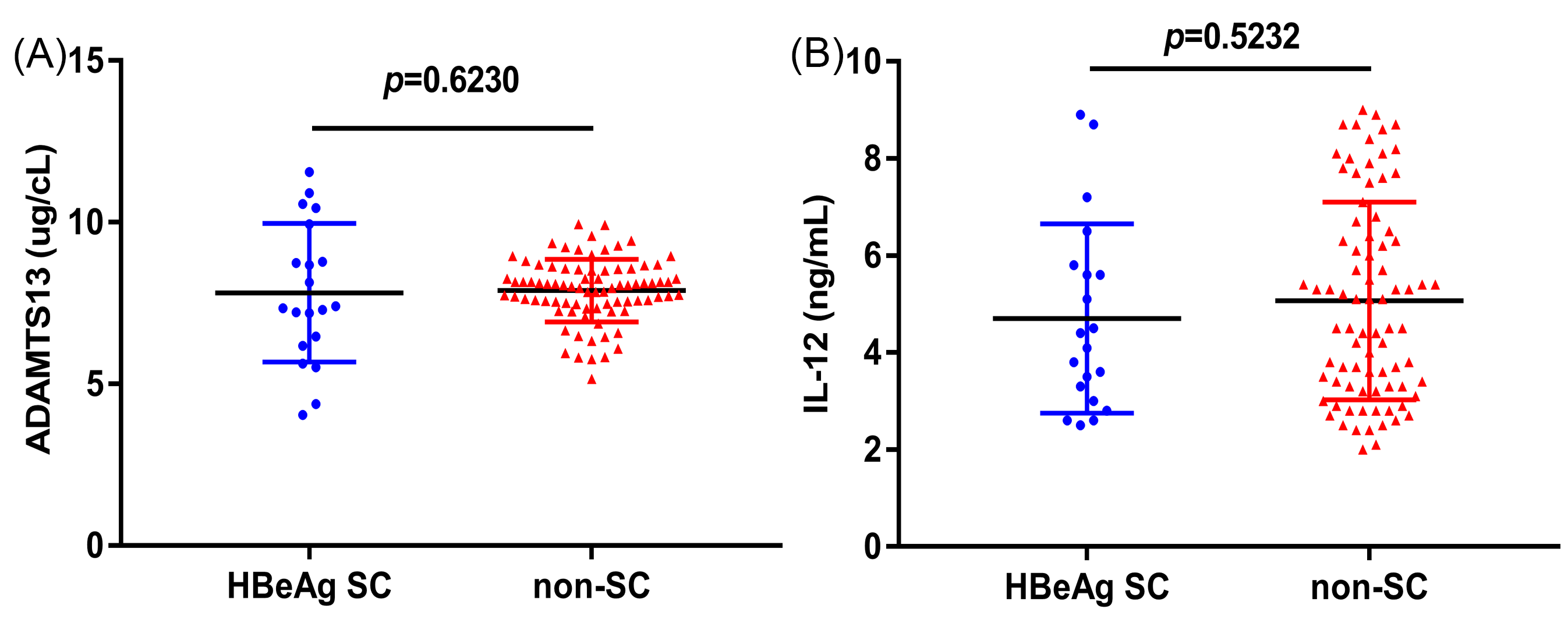

Supplement: Figure S1 — Comparison between CHB patients with and without HBeAg seroconversion in plasma ADAMTS13 and IL-12 levels during m-ETV treatment. Plasma ADAMTS13 (A) baseline levels are no significantly difference in those CHB patients with or without HBeAg SC, similarly, the IL-12 (B) baseline levels are no significantly difference either. [file Image_1.TIF]

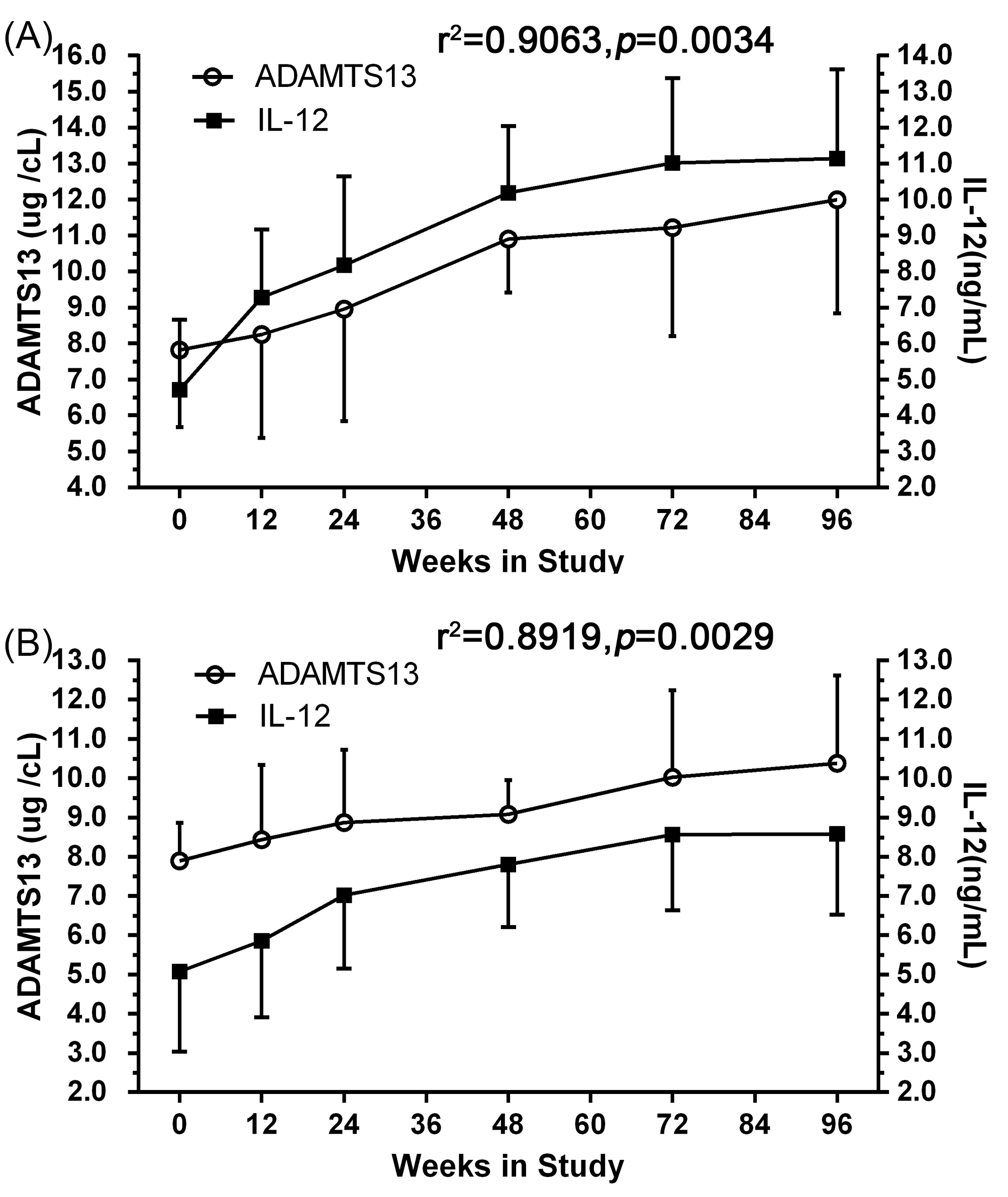

Supplement: Figure S2 — Association between plasma ADAMTS13 and IL-12 levels in HBeAg seroconverting (A) and non-seroconverting (B) patients during m-ETV treatment. [file Image_2.TIF]

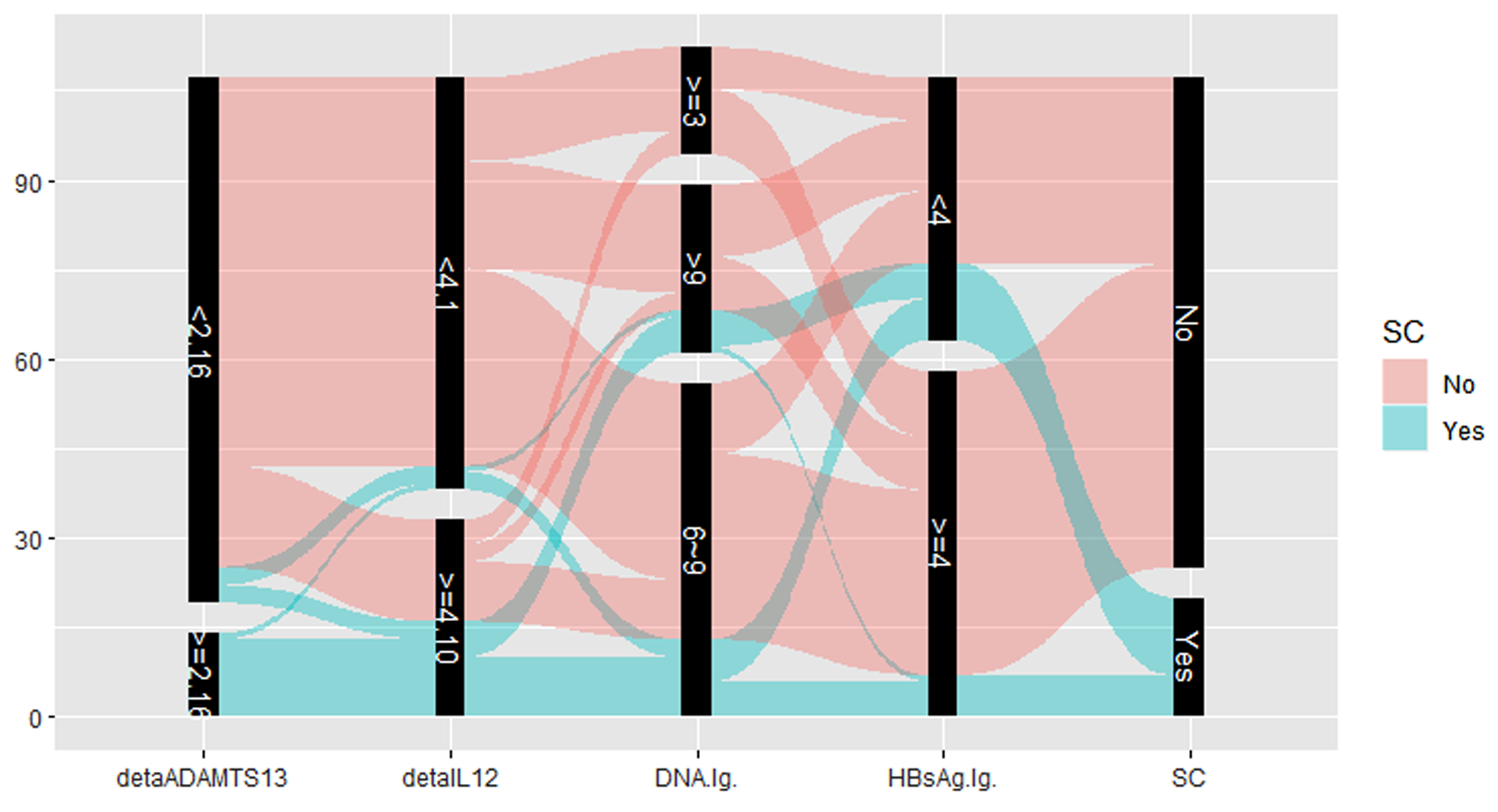

Supplement: Figure S3 — Sankey diagram of predicting HBeAg seroconversion. The variables of ΔADAMTS13, ΔIL-12, DNA (lg), HBsAg (lg), and HBeAg seroconversion (Yes, No) were put together to form Sankey diagram, in which variables could vividly present their contributing rate for SC in CHB patients during m-ETV treatment. The X-axis (left vertical numbers) shows the approximate number of CHB patients. [file Image_3.TIF]

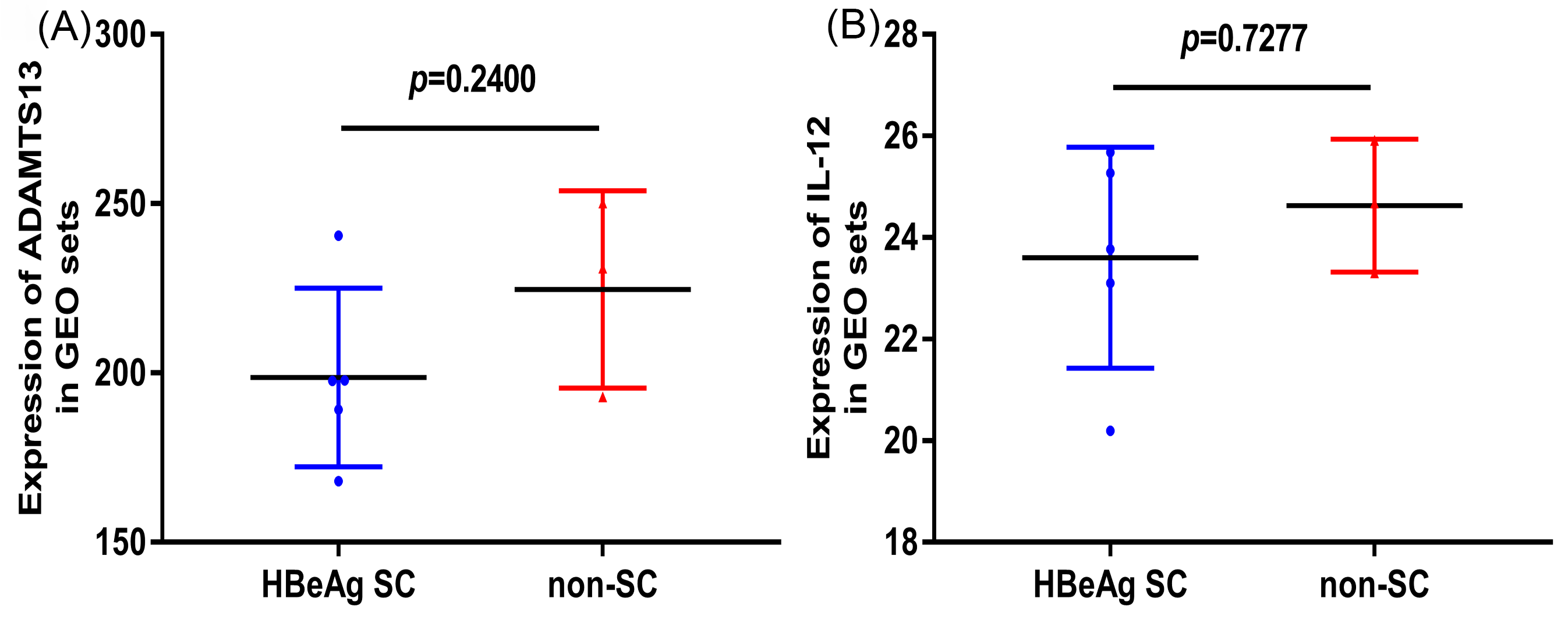

Supplement: Figure S4 — Expression of ADAMTS13 (A) or IL-12 (B) levels between patients with and without HBeAg seroconversion (SC). The data is from the GEO dataset (GSE54747), and was grouped into two groups (HBeAg SC and non-SC HBeAg) according to the message provided. Dataset (https://www.ncbi.nlm.nih.gov/geo/query/acc.cgi?acc=GSE54747). [file Image_4.TIF]
